# 

**Published:** 1872-02

**Authors:** 


### CONTENTS:

ORIGINAL COMMUNICATIONS:

ART. I. Clinical Lectures on Diseases of the Ear. By E. L. Holmes, M.D., Prof. Ophthalmology, etc., in Rush Med. Col., and Surgeon to Chicago Charitable Eye and Ear Infirmary.. 65

ART. II. St. Luke's Hospital. Oblique fracture of femur at junction of middle and lower thirds; union without shortening; anchylosis from adhesions at seat of fracture. Oblique fracture of femur at junction of middle and upper thirds; union with three-fourths inch shortening. (Under care of John E. Owens, M.D.)........... 69

SELECTIONS ............................ 71

EDITORS' BOOK TABLE................. 114

EDITORIAL............................ 116

ITEMS, NEWS AND GOSSIP.............. 117

LOOT ................................ 120

